# Direct binding of hepatocyte growth factor and vascular endothelial growth factor to CD44v6

**DOI:** 10.1042/BSR20150093

**Published:** 2015-08-07

**Authors:** Yvonne Volz, David Koschut, Alexandra Matzke-Ogi, Marina S. Dietz, Christos Karathanasis, Ludovic Richert, Moritz G. Wagner, Yves Mély, Mike Heilemann, Hartmut H. Niemann, Véronique Orian-Rousseau

**Affiliations:** * Karlsruhe Institute of Technology (KIT), Institute of Toxicology and Genetics (ITG), Postfach 3640, 76021 Karlsruhe, Germany; † Institute for Physical and Theoretical Chemistry, Goethe-University Frankfurt, Max-von-Laue-Str. 7, 60438 Frankfurt, Germany; ‡ UMR 7213 CNRS, Laboratoire de Biophotonique et Pharmacologie Faculté de Pharmacie 74 route du Rhin, 67401 Illkirch, France; § Agilent Technologies, Hewlett-Packard-Straße 8, 76337 Waldbronn, Germany; ║ Department of Chemistry, Bielefeld University, Universitätsstr. 25, 33615 Bielefeld, Germany; ¶ amcure GmbH, Hermann-von-Helmholtz Platz 1, 76344 Eggenstein-Leopoldshafen, Germany

**Keywords:** binding affinity, CD44s, CD44v6, HGF, Met, VEGF

## Abstract

CD44v6, a member of the CD44 family of transmembrane glycoproteins is a co-receptor for two receptor tyrosine kinases (RTKs), Met and VEGFR-2 (vascular endothelial growth factor receptor 2). CD44v6 is not only required for the activation of these RTKs but also for signalling. In order to understand the role of CD44v6 in Met and VEGFR-2 activation and signalling we tested whether CD44v6 binds to their ligands, HGF (hepatocyte growth factor) and VEGF (vascular endothelial growth factor), respectively. FACS analysis and cellular ELISA showed binding of HGF and VEGF only to cells expressing CD44v6. Direct binding of CD44v6 to HGF and VEGF was demonstrated in pull-down assays and the binding affinities were determined using MicroScale Thermophoresis, fluorescence correlation spectroscopy and fluorescence anisotropy. The binding affinity of CD44v6 to HGF is in the micromolar range in contrast with the high-affinity binding measured in the case of VEGF and CD44v6, which is in the nanomolar range. These data reveal a heparan sulfate-independent direct binding of CD44v6 to the ligands of Met and VEGFR-2 and suggest different roles of CD44v6 for these RTKs.

## Abstract (precis)

CD44v6 is a co-receptor for the receptor tyrosine kinases Met and VEGFR-2 (vascular endothelial growth factor receptor 2). The binding of these RTKs (receptor tyrosine kinases) to their ligands on cells requires CD44v6. Pull-downs assays show direct binding between these entities. Binding affinities were measured by several biophysical methods.

## INTRODUCTION

Receptor tyrosine kinases (RTKs) are single-pass transmembrane proteins with an extracellular ligand binding domain and an intracellular catalytic domain. They are thought to get activated by ligand-induced dimerization (or more generally oligomerization) of their ectodomains, facilitating trans-phosphorylation of their tyrosine kinase domains. However, increasing evidence show that the activation process of RTKs is not only dependent on ligand binding but requires in addition the contribution of cell adhesion molecules (CAMs) (reviewed in [1]). These CAMs act in most cases as co-receptors providing a plethora of functions to the RTKs. They participate in the activation process by clustering the ligand thereby facilitating its binding to RTKs or by promoting dimerization. They also take part in the signalling process by amplifying the signals emanating from the RTKs and even participate in the internalization process.

The first described co-receptors for RTKs are the heparan sulfate proteoglycans (HSPGs) that act as low-affinity receptor for the fibroblast growth factor receptor (FGFR). These low-affinity receptors foster clustering of growth factors thereby concentrating the ligands in the vicinity of the high affinity receptor. The studies of Yayon et al. [2] demonstrated a requirement of heparin/HS for FGFR activation. Additional data showed that the FGF-2 (fibroblast growth factor 2)/FGFR-1 interaction as well as the FGF-2-induced signalling is stimulated by HSPGs such as syndecans and glypicans [3,4]. Syndecan-2 on human macrophages stimulates FGF-2-mediated proliferation [5], and glypican-1 activates FGF-2 signalling [6,7]). HSPGs stabilize the FGF:FGFR dimers [8]. Heparin/heparan sulfate (HS) binds to a positively charged canyon formed by the two FGF receptors associated in the dimer and the adjoining bound FGF molecules [9].

In the case of both VEGFR-2 (vascular endothelial growth factor receptor 2) and Met, prominent RTKs involved in angiogenesis and metastasis, CD44v6 is one of the best characterized co-receptors (reviewed in [10]). CD44v6, a non-heparin/HS sulfated member of the CD44 family of transmembrane glycoproteins [11] is required for Met and VEGFR-2 activation and signalling in several cancer cell lines and primary cells. More precisely, the ectodomain of CD44v6 takes part in the activation process whereas the cytoplasmic domain of CD44 is involved in signalling (reviewed in [12]). CD44v6 controls Met internalization and is also co-internalized with Met upon induction by HGF (hepatocyte growth factor) [13]. Ala-scan mutation of the CD44v6 exon sequence revealed the importance of three amino acids in the v6 exon sequence for the co-receptor function of CD44v6 for Met [14]. Mutation of these sequences corresponding to EWQ in rat, RWH in human and GWQ in mouse CD44v6 prevented Met activation. Small peptides (v6 pep) ranging from 5 to 14 amino acids covering these three essential amino acids inhibited Met and VEGFR-2 activation and signalling, and consequently tumour angiogenesis [15].

VEGFR-2 also recruits another co-receptor, namely neuropilin (NRP-1/2), thereby promoting a full-blown angiogenic response (reviewed in [16]). In that case, VEGF (vascular endothelial growth factor) is able to bridge VEGFR-2 to NRP-1/2 by binding both molecules simultaneously. Neuropilin is a low-affinity receptor for VEGF that has essential functions in VEGF-induced angiogenesis. NRP-1 also takes part in the trafficking of VEGFR-2 thereby controlling signalling [17]. Indeed, association of VEGFR-2 with NRP-1 induces p38 MAPK signalling. CD44v6 is therefore an additional co-receptor of VEGFR-2 that is essential for the activation process and signalling in several primary endothelial cells [15]. Its precise molecular mechanism of action is still elusive.

Many open questions also remain concerning the activation process of Met and the exact molecular contribution of CD44v6. Upon HGF induction a ternary complex between Met, HGF and CD44v6 could be detected in several cell lines [13,18,19].

The interactions between HGF, Met and CD44v6 on the one hand and VEGF, VEGFR-2 and CD44v6 on the other hand need to be further characterized. In the present paper, using FACS and cellular ELISA we show binding of HGF and VEGF only to cells expressing CD44v6. Furthermore, pull-down assays reveal a direct binding between these entities. The binding affinities of purified CD44v6 ectodomain for VEGF and HGF respectively were measured using MicroScale Thermophoresis (MST), fluorescence correlation spectroscopy (FCS) and fluorescence anisotropy (FA).

## EXPERIMENTAL

### Cells

Human umbilical vein endothelial cells (HUVEC; PromoCell, Heidelberg, Germany) were grown in Endothelial Cell Growth Medium complemented with Supplement Mix (PromoCell). The human embryonic kidney cells (HEK293T; ATCC, Wesel, Germany), the human colon adenocarcinoma cells (HT29), a gift from Alain Zweibaum (Institute National de la Sante et de la Recherche Medicale, Paris, France) and the human cervix carcinoma cells (HeLa; ATCC) were grown in Dulbecco's Modified Eagle's Medium (DMEM; Invitrogen, Karlsruhe, Germany) supplemented with 10% FBS (FBS Gold; PAA Laboratories). The rat pancreatic carcinoma cells BSp73AS (AS) and its transfectant BSp73ASs6 (ASs6) were already described [18] and grown in RPMI (Invitrogen) plus 10% FBS. The human ductal breast epithelial tumour cell lines (T47D and T47D/Met/T47D2A) were a gift from Morag Park (McGill University, Montreal, Canada) and cultured in DMEM supplemented with 10% FBS.

### Reagents

The antibodies used in the present study were directed against rat CD44v6 (1.1ASML, described in [20]) and human CD44v6 (VFF18; Boehringer Ingelheim), Erk-1 (K-23; Santa Cruz), phospho-p44/42 MAPK (Thr202/Tyr204; Cell Signaling Technology), penta-His (Qiagen), human HGF (AF-294-NA; R&D Systems), rat Met (B2; Santa Cruz), human Met (25H2; Cell Signaling Technology) phospho-Met (Tyr1234/1235; Cell Signaling Technology), human TGF-α (transforming growth factor alpha; Peprotech), VEGFR-2 (A3; Santa Cruz) and human VEGF-A165 (VEGF_165_) (AF-293-NA; R&D Systems). The secondary antibodies labelled with horseradish peroxidase (HRP) were from Dako, the phycoerythrin (PE)-labelled antibody from Thermo Scientific and the FITC–streptavidin from Invitrogen. The human HGF was a generous gift from Ermanno Gherardi (University of Pavia, Pavia, Italy). Human VEGF_165_ and human TGF-α were from Peprotech. The peptides have already been described in [14] and their sequences are: rat CD44v6 5mer–NEWQG, rat CD44v6 14mer–KEKWFENEWQGKNP, human CD44v6 5mer–NRWHE, human CD44v6 14mer–KEQWFGNRWHEGYR, mouse CD44v6 5mer–NGWQG and mouse CD44v6 14mer–QETWFQNGWQGKNP.

### Constructs

The rat CD44v6 expression construct used for protein expression and purification of the CD44v6 ectodomain was kindly provided by Kurt Ballmer-Hofer (Paul Scherrer Institute, Villigen, Switzerland). Here the extracellular part of the rat CD44v6 sequence was cloned in-frame with a hexahistidine sequence at the C-terminal end of the CD44 sequence into a pcDNA3.1(+) plasmid. The human CD44s ectodomain was cloned in the same way into the pcDNA3.1(+) vector.

The human Met_928_ construct, comprising almost the entire extracellular sequence of Met, was expressed in CHO Lec8 cells. The cells were cultured in FCS-free medium and secreted the C-terminally His-tagged Met_928_ protein into the medium for the following NiNTA superflow (Qiagen) affinity chromatography protein purification. The conditions have been described in detail in [21,22].

The VEGFR-2 expression construct has been previously described [15]. The VEGFR-2 expression plasmid pBE hVEGFR-2 was derived from the pEGFP-C1 vector from Clontech by introducing the sequence encoding the human VEGFR-2 with the PCR subcloning method to remove the GFP reading frame with the forward primer: GCTCTTCGGGGAGCAGCGATGGAGAGCAAGGTGCTGCTG and the reverse primer: GGAGGTTTTTTAAAGCAAGTAAAACCTTTATCACAGATCCTCTTCTGAGATGAG.

The expression vector secreting the soluble part of CD44 (either CD44s or CD44v4–7Δ15) used in the FACS experiments has been described in Orian-Rousseau et al. [18]. In brief, a CD44s or a CD44v4-v7Δ15-human Fc fusion was cloned in the CDM8 vector.

### Expression and purification of CD44 proteins

The CD44 constructs (rat CD44v6 and human CD44s) were transiently transfected in HEK293T cells using 20 μg/ml branched polyethylenimine (Sigma-Aldrich). Two days after transfection sodium butyrate was added to a final concentration of 6 mM. Four days post transfection the supernatant was collected and concentrated via a 10 kDa cut off membrane (Sartorius) using a Sartocon ultrafiltration device (Sartorius) and diluted 1:10 in loading buffer (50 mM HEPES, 260 mM NaCl and 10 mM imidazole, pH 7) and again concentrated via ultrafiltration. The proteins were purified on a nickel-nitrilotriacetic acid (Ni-NTA) column (20 ml HisPrep FF 16/10 column; GE Healthcare) and eluted with a linear gradient of an elution buffer (50 mM HEPES, 260 mM NaCl and 1 M imidazole, pH 7). The fractions from purification were pooled, concentrated and rebuffered in storage buffer (50 mM HEPES, 150 mM NaCl and 5% glycerol, pH 7.5) via ultrafiltration (Vivaspin 10 kDa cut off; Sartorius). Proteins were then subjected to Western blot analysis.

### Activation of Met and Erk

ASs6 cells were serum-starved for 24 h, and then induced with 20 ng/ml HGF for 5 min at 37°C. Where indicated, the cells were treated with different concentrations of CD44s ectodomain and CD44v6 ectodomain for 5 min at 37°C prior to induction. Activated Met and Erk was determined using Western blot analysis as previously described [15].

### Fluorescence-activated cell sorting

Binding of human HGF and human VEGF_165_ to cells was measured by FACS analysis. Cells were seeded at a density of 5×10^5^ cells in 5 cm plates. For the HGF binding assay in presence of the CD44v6 and CD44s ectodomain, the T47D and T47D/Met cells were transfected with the corresponding expression vectors using Lipofectamine (Invitrogen) according to the manufacturer's protocol. On the next day cells were serum-starved for 24 h. For blocking experiments serum-starved AS, ASs6, T47D and T47D/Met cells were incubated either with the respective species-specific CD44v6 antibody (100 μg/ml), v6 or control peptide (100 ng/ml) for 1 h at 4°C and then induced with 20 ng/ml biotinylated HGF for 1 h at 4°C. Biotinylation of HGF was performed as described in [18]. After two washing steps using PBS (Cat. #14190; Invitrogen) the cells were detached from the plates using PBS supplemented with 5 mM EDTA and resuspended in PBS containing 3% FBS. The cells were incubated with FITC–streptavidin (1 μg/ml) for 30 min on ice followed by three final washing steps and resuspension in PBS. The fluorescence was analysed in a FACScan flow cytometer (Becton Dickinson). For the VEGF binding assay serum-starved HUVEC and ASs6 cells were incubated with the respective species-specific v6 or control peptide as described above followed by induction with 20 ng/ml of VEGF_165_ for 1 h at 4°C. After washing and detaching the cells were incubated with an antibody against VEGF_165_ (10 μg/ml) for 1 h at 4°C. Following three washing steps the cells were incubated with an anti-goat IgG (PE-conjugate) for 30 min at 4°C protected from light, washed, resuspended in PBS and analysed in the FACScan flow cytometer.

### Cellular enzyme-linked immunosorbent assay

Cells were seeded in 96-well plates at a concentration of 4x10^4^ cells per well. AS and ASs6 cells were transfected with a VEGFR-2 expression vector using FuGene (Promega) according to the manufacturer's protocol. On the next day cells were serum-starved for 24 h. After starvation cells were washed twice with PBS and blocked for 1 h at room temperature with 100 μl/well of PBS plus 5% BSA. Where indicated, cells were treated with 100 ng/ml rat v6 peptide or 100 ng/ml control peptide for 1 h at 4°C prior to the induction with 20 ng/ml biotinylated human HGF, 20 ng/ml biotinylated human VEGF_165_ (as performed for HGF) or 20 ng/ml biotinylated soybean trypsin inhibitor (used as negative control) (R&D Systems) for 1 h on ice. The cells were then incubated with an avidin–HRP conjugate (Abcam) for 30 min at 4°C protected from light. Cells were washed twice with PBS and the ABTS solution (Roche Diagnostics) was added for 45 min at room temperature. The absorption at 415 nm was determined with an ELISA reader EXL 808 (Biotek). Experimental data of the negative controls (biotinylated soybean trypsin inhibitor) were directly subtracted from the experimental data of the biotinylated human HGF and VEGF_165_.

### Liquid chromatography time-of-flight spectrometry

CD44v6 was digested with trypsin and analysed using ultra-high performance liquid chromatography tandem mass spectrometry (UHPLC–MS/MS, Agilent Technologies). 2 μl of each sample (corresponding to 1 μg) were injected. The chromatographic separation was performed using reverse-phase chromatography with water plus 0.1% formic acid as solvent A and acetonitrile plus 0.1% formic acid as solvent B. The gradient consisted of an isocratic step for 0.5 min at 3% solvent B, followed by linear gradients from 3% to 60% solvent B over 5.5 min and from 60% to 100% B over 1 min. The column was washed at 100% B for 1 min followed by a linear gradient from 100% to 3% B within 0.1 min and 1.9 min re-equilibration time. The overall running time was 10 min at a flow rate of 0.5 ml/min and the column was thermostatted at 20°C. The quadrupole-time-of-flight mass spectrometer was tuned and calibrated in 2 GHz “extended dynamic range mode” for a mass range up to 3200 *m*/*z* and operated in data-dependent “Auto MS/MS” mode. Positive electrospray ionization was carried out using a capillary voltage of 4500 V and a fragmentor voltage of 175 V. Nitrogen was used as drying gas with 8 l/min at 300°C and sheath gas respectively with 11 l/min at 350°C, nebulizer pressure was 35 psig. Data-dependent data acquisition was carried out using “Auto MS/MS” mode. Alternating acquisitions in MS and MS/MS were carried out detecting both intact peptide masses and peptide fragment masses respectively after collision-induced dissociation (CID) using nitrogen as a collision gas. The scan rate for both MS and MS/MS was set to 5 Hz, the collision energy was ramped depending on precursor *m*/*z* with an offset of 3 V and a slope of 4 V/100 Da (Dalton). The precursor charge state preference was 2 > 3 > 4 or higher > 1 > unknown. A maximum of 2 precursors was isolated and fragmented within each cycle using both dynamic exclusion after 2 spectra and release after 0.5 min and static exclusion for precursors with 100–300 *m*/*z* and 900–3200 *m*/*z*.

Data interpretation was carried out by matching the obtained MS and MS/MS spectra against the hypothetical “in-silico-digest” of CD44v6 protein sequence. Herewith, no missed cleavage and post-translational modification such as glycations were taken into consideration and only masses within a mass tolerance of 10 ppm for MS and 50 ppm for MS/MS were accepted.

### Scattering assay

Cells were seeded in 24-well plates at a density of 4×10^4^ cells per well and serum-starved the next day for 24 h. Where indicated 25 μg/ml human CD44s ectodomain or 25 μg/ml rat CD44v6 ectodomain were applied and incubated for 5 min at 37°C prior to induction with 20 ng/ml HGF. 24 h later cells were observed with the Axiovert 40c Zeiss microscope (10× objective, Carl Zeiss AG) and pictures were taken using a PowerShot S620 digital camera (Canon).

### *In vitro* monolayer wound closure assay

Cells were seeded in 12-well plates at a density of 1.5×10^5^ cells per well and serum-starved the next day for 24 h. After starvation a scratch was made into the confluent cell layer using a sterile pipette tip. The medium was changed and replaced by fresh medium or medium containing 25 μg/ml human CD44s ectodomain or 25 μg/ml rat CD44v6 ectodomain. After 5 min at 37°C, induction with 20 ng/ml HGF was performed. Pictures were taken directly after the scratch and 24 h after induction using a PowerShot S620 digital camera (Canon). The computer program ImageJ (National Institutes of Health) was used for quantitative evaluation. The area covered by cells that migrated into the wound was quantified.

### Nickel pull-down assay

For the pull-down assays Ni-NTA beads (Qiagen) were used. 30 μl of the Ni-NTA beads were washed once in pull-down (PD) buffer (50 mM HEPES, 150 mM sodium chloride and 100 mM imidazole, pH 7.5) and resuspended in it. 0.5 μg of the ectodomains were added together with 0.5 μg of the different growth factors (HGF, VEGF_165_ and TGF-α) and incubated on a spinning wheel for 2 h at 4°C. After incubation the beads were washed three times with the PD buffer, the supernatant was removed and the nickel-bound proteins were subjected to Western blot analysis.

### MicroScale Thermophoresis

10 μM of HGF, VEGF_165_ and CD44v6 ectodomain were labelled with a red fluorescent dye (NT-647) using the Protein Labeling NHS RED Kit (NanoTemper Technologies). 10 μM of proteins were incubated with 30 μM of the fluorophore NT-647 for 30 min at room temperature protected from light. Meanwhile a gravity flow column was equilibrated with 10 ml PBS. After incubation the labelled protein was loaded on the gel filtration column to separate free dye from labelled protein. 10 nM of fluorescently labelled protein (HGF, VEGF_165_ and CD44v6 ectodomain) was added to a serial dilution of unlabelled protein (serial dilutions: 0.8 nM to 27 μM of CD44v6 or CD44s, 0.4 nM to 7 μM of Met_928_ and 2.1 nM to 70 μM of TGF-α and 0.1 nM to 10 μM of CD44 v6 peptide). The samples were loaded into hydrophilic capillaries (NanoTemper Technologies, reference K004). Measurements were performed in the Monolith NT.115 at 22°C in PBS plus 1% BSA by using 50% LED power and 80% IR-laser power. Data were analysed using NanoTemper Analysis software v.1.4.23 and plotted using the OriginPro v.8.6 software from OriginLab.

### Fluorescence correlation spectroscopy

The FCS measurements were performed using either a commercial ConfoCor2 fluorescence correlation spectrometer (Carl Zeiss AG) or a custom-built confocal setup. The custom-built setup was equipped with a 640 nm diode laser (Cube 640; Coherent). The laser beam was reflected into a water immersion objective (UPLSAPO, 60x, NA 1.2; Olympus) by a dichroic mirror (650DCXR; AHF Analysentechnik). The emission light passed the dichroic mirror and was focused with an achromatic lens (*f*=100 mm) onto a pinhole (100 μm). A second lens (*f*=100 mm) parallelized the light again. Passing a bandpass filter (700/75 ET, AHF Analysentechnik), the emission light was separated by a 50/50 beamsplitter (AHF Analysentechnik). Two convex lenses (*f*=25.5 mm) focused the emission light on to the active area of two single-photon APDs (SPCM-AQRH-14; PerkinElmer). A Flex03LQ correlator card (correlator.com) cross-correlated the fluorescence signal. In the ConfoCor2, the 633 nm laser was used to excite the fluorophores in the sample. A water immersion objective (40×, NA 1.2 C-Apochromat) focused the laser light into the sample.

HGF, VEGF_165_ and TGF-α were labelled with ATTO 647N NHS ester (ATTO-TEC) according to the manufacturer's protocol. For FCS experiments, samples were prepared in PBS buffer containing 0.05% Tween-20 (PBST). Additionally, a protocol using poly-L-lysine polyethylene glycol (PLL-PEG) was established to prevent glass adsorption. Here, coverslips (Ø 19 mm) were cleaned for 20 min by sonification in isopropyl alcohol, washed in water, and dried with nitrogen. After 10 min plasma cleaning, 10 μl 1 mg/ml PLL-PEG in bidistilled water were dropped on to a coverslip and a second coverslip was placed on top of it. After 1.5–2 h incubation, coverslip sandwiches were separated in bidistilled water and dried with nitrogen.

For sample preparation, the unlabelled binding partner was added in different concentrations in the range from 0.1 nM to 20 μM to the fluorescently labelled protein (1–5 nM). The complex formation was allowed to occur for at least 3 h at room temperature. Each measurement was done at a constant imaging depth of 80 μm (custom-built setup) or 200 μm (ConfoCor2) from the glass surface. For each measurement, the fluorescence signal was recorded for 5 min. All measurements were carried out at 20°C.

Diffusion times were determined by fitting with a 2D diffusion model as described elsewhere [23]. Complete binding curves were determined by plotting the diffusion times at different concentrations of the unlabelled protein. Fitting with a 1:1 binding model allows estimation of the dissociation constant *K*_d_.

### Fluorescence anisotropy

A spectrofluorimeter (SLM 8000, SPEX, USA) in the T-format configuration was used to measure steady-state FA (*r*). The excitation wavelength was set at 580 nm for HGF tagged with the red fluorescent dye (NT-647) using the Protein Labeling NHS RED kit (NanoTemper Technologies) as described for MST. The emitted light was monitored with a 600 nm long pass filter (Wratten 29, Kodak). The labelled HGF protein was titrated with increasing concentrations of CD44v6 or CD44s. A home-built device ensured the automatic rotation of the excitation polarizer, allowing continuous measurement of the anisotropy during 120 s. Temperature was fixed at 20°C. Anisotropy values were calculated and recorded with the Bio-Kine program (Bio-Logic). As the intensity of fluorescence of the labelled HGF was found to be not affected by the binding process, the anisotropy *r* of HGF of can be expressed by:

$$r\;=\;r_{f}\;+\;\frac{r_{b}-r_{f}}{n}\times \left(\frac{\left(1+Ka\times \left(n\times P_{t}+L_{t}\right)\right)-\sqrt{{\left(1+Ka\times \left(n\times P_{t}+L_{t}\right)\right)}^{2}-\left(4\times Ka^{2}\times n\times P_{t}\times L_{t}\right)}}{2\times Ka\times L_{t}}\right)$$

where *L_t_* and *L* are respectively the total and free concentrations of CD44v6 or CD44s, *P_t_* is the total concentration of HGF, *n* is the binding stoichiometry of the complex, *r_f_* and *r_b_* are the anisotropy of the free and fully bound HGF protein, respectively.

## RESULTS

### Binding of HGF and VEGF to cells detected only in CD44v6-expressing cells

**Figure 1:**
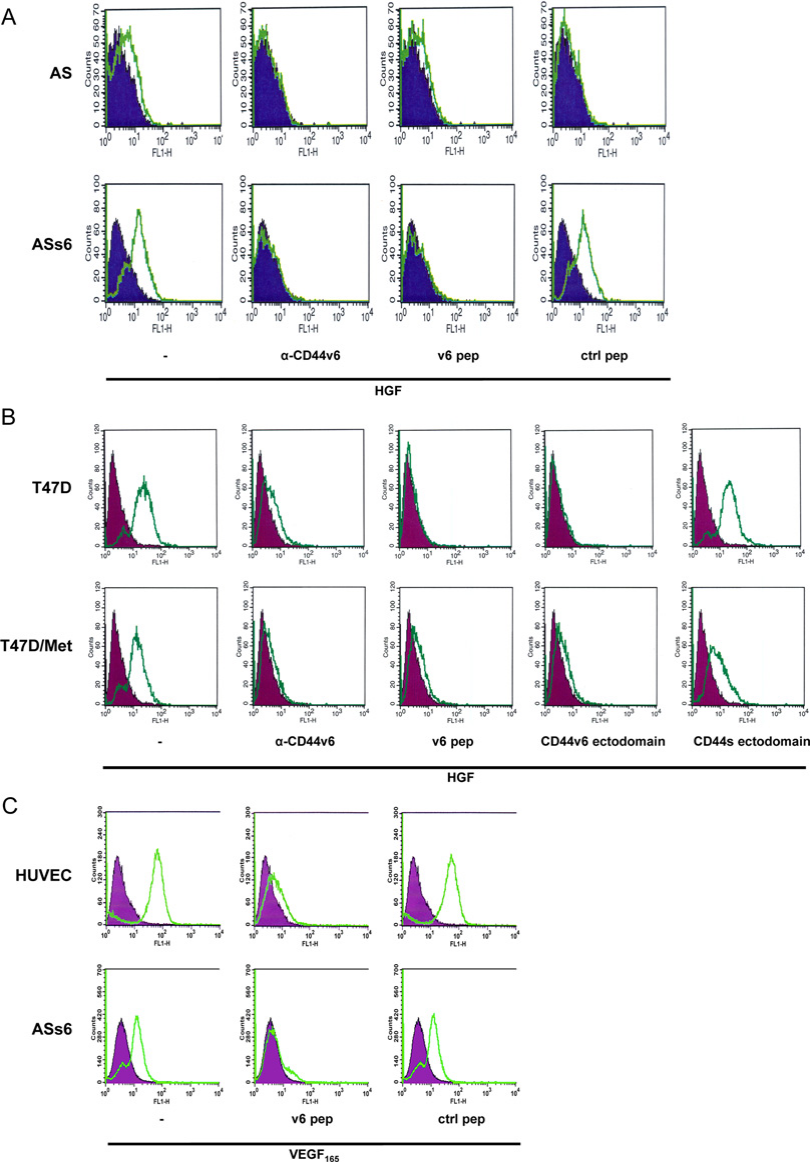
CD44v6 is required for binding of HGF and VEGF to cells (**A**) Serum-starved Bsp73AS (AS) or Bsp73ASs6 (ASs6) cells were incubated with a CD44v6 antibody (1.1ASML) [20], a v6 peptide (14mer) [14] or a control peptide prior to induction with biotinylated HGF and subsequent detection of binding using FITC–streptavidin. All samples were analysed in a FACScan flow cytometer. The FITC–streptavidin negative control is shown in blue, HGF-induced samples either non-treated or treated with CD44v6-specific blocking reagents are shown in green. Representative pictures from one experiment are shown. Three independent experiments per condition were performed giving the same results. (**B**) Serum-starved T47D and T47D/Met cells were pretreated either with a CD44v6 antibody (VFF18), or a v6 peptide (14mer) prior to induction with biotinylated HGF. Expression of CD44v6 and CD44s.ectodomains (labelled as Ecd) was achieved by transfection of the expression constructs using Lipofectamine. The cells were then subjected to FACS analysis 48 h after transfection. Binding of biotinylated HGF was detected using FITC labelled streptavidin. All samples were analysed in a FACScan flow cytometer. Cells exclusively incubated with FITC–streptavidin were used as a negative control (purple). Cells treated with HGF alone, or pretreated with the peptides, antibody or transfected with the ectodomains are represented in green. Representative pictures from one experiment are shown. Three independent experiments were performed giving the same results. (**C**) Serum-starved HUVEC and ASs6 cells were pre-incubated either with a v6 peptide (5mer) [14] or a control peptide as indicated and induced with VEGF_165_. Binding of VEGF_165_ to the cells was detected by means of a specific antibody against VEGF and a secondary PE-labelled antibody and subsequently analysed in a FACScan flow cytometer. Cells exclusively incubated with the PE-labelled antibody were used as a negative control (purple). Cells treated with VEGF_165_ or additionally pre-incubated with the peptides and probed with the antibodies are represented in green. Representative pictures from one experiment are shown. Three independent experiments were performed giving the same results.

Since the CD44v6 ectodomain is required for Met activation (reviewed in [12]), we hypothesize that its role in Met activation is linked to its ability to bind HGF. In order to test whether CD44v6 is involved in the binding of HGF to Met-positive cells, we performed FACS analyses. Binding of biotinylated HGF to BSp73ASs6 (ASs6) cells that express CD44s, the smallest CD44 isoform, CD44v6 and Met (Supplementary Figure S1A) and in which Met can be activated [14] was measured by FACS using streptavidin. The Bsp73AS (AS) cells that express Met and CD44s but do not express CD44v6 ([14]; Supplementary Figure S1A) were used as control. In these control cells, Met activation was either low or undetectable. The FACS analysis showed that HGF bound to ASs6 cells but not to AS cells (Figure 1A). This binding was dependent on CD44v6 as blocking reagents such as the CD44v6 antibody (α-CD44v6) or a CD44v6 specific peptide (v6 pep) completely abrogated this binding. In contrast, a control peptide (ctrl pep) had no effect.

Since binding of HGF to cells could only be observed in cells expressing CD44v6, we investigated whether CD44v6 alone might be sufficient for this binding. We compared the binding of biotinylated HGF to T47D cells that do not express Met but express CD44v6 ([24] and Supplementary Figure S1B) to T47D/Met cells that express both proteins (Figure 1B). As expected, HGF bound to cells that express both CD44v6 and Met. This binding was specific to CD44v6 as it could be blocked by transfection of a CD44v6 ectodomain whereas transfection of a CD44s ectodomain had no effect. Binding of HGF to T47D/Met cells was also blocked by means of a CD44v6 antibody (α-CD44v6) and the v6 peptide. Surprisingly however, binding of HGF was also observed in the absence of Met in T47D cells. Again, this binding was inhibited upon transfection of the CD44v6 ectodomain as well as pre-incubation with the CD44v6 antibody and the v6 peptide but not by transfecting the CD44s ectodomain (Figure 1B).

CD44v6 can also act as a co-receptor for VEGFR-2 [15]. Therefore, similar experiments were performed using VEGF_165_, a member of the VEGF family inducing the VEGFR-2 receptor. Using FACS, we tested whether the binding of VEGF_165_ to

VEGFR-2 positive cells was dependent on CD44v6 using an antibody against VEGF (Figure 1C). HUVECs that express CD44v6 [15] and VEGFR-2 were used. As expected, VEGF_165_ could bind to HUVECs and this binding could be blocked by means of the v6 peptide whereas the ctrl peptide had no effect (Figure 1C). Interestingly, VEGF_165_ was also able to bind to ASs6 cells (Figure 1C) that do not express VEGFR-2 but express CD44v6 (Supplementary Figure S1C).

**Figure 2:**
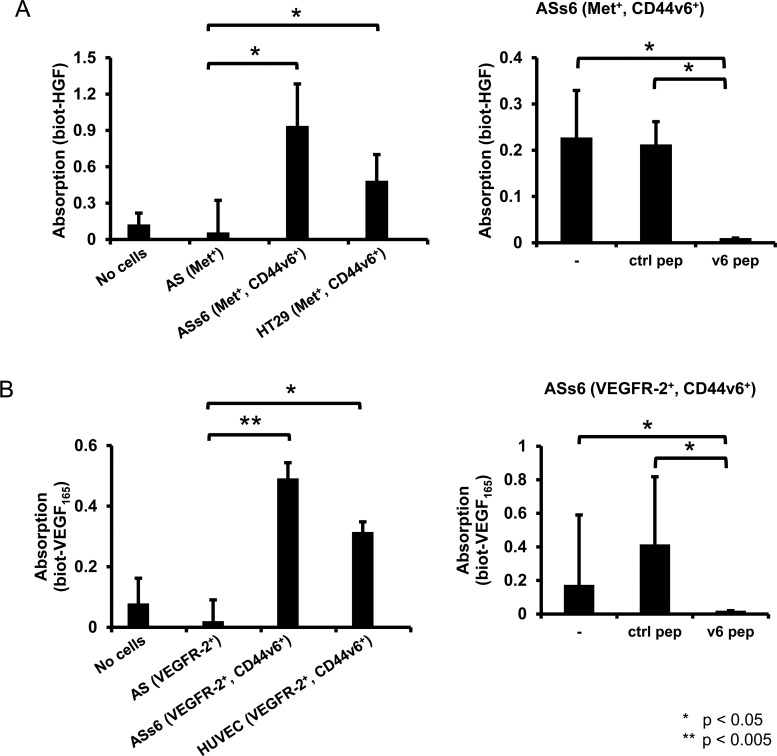
CD44v6 is required for binding of HGF and VEGF to cells (**A**) For the cellular ELISA serum-starved AS, ASs6 and HT29 cells were induced with biotinylated HGF. The binding of the growth factor to the cells was detected by means of an avidin–HRP conjugate and ABTS solution and subsequently analysed in an ELISA reader. Experimental data are reported as mean ± S.D. of three independent experiments (*P< 0.05, **P< 0.005; Student's *t*-test). ASs6 cells were in addition treated with a v6 (5mer) and a control peptide as indicated and induced with biotinylated HGF. (**B**) The AS and ASs6 cells were transfected with a VEGFR-2 expression construct using FuGene. 48 h after the transfection the cellular ELISA was performed with AS, ASs6 and HUVEC cells as described above and the cells were induced with biotinylated VEGF_165_.

To further study the involvement of CD44v6 in the binding of HGF to cells, we performed a cellular ELISA. In this experiment, AS, ASs6 or HT29 cells were seeded into 96-well plates (Figure 2A, left panel). The binding of biotinylated HGF to these cells was monitored using an avidin–HRP conjugate. A clear binding of HGF to ASs6 cells expressing Met and CD44v6 was observed. In contrast, no binding of HGF to cells expressing Met but no CD44v6 (AS) could be detected. The control wells were not coated with cells and the unspecific binding of HGF to the plastic surface was evaluated. HGF could bind to HT29 cells that were used as a positive control. Indeed, in HT29 cells that express both CD44v6 and Met, the co-receptor function of CD44v6 for Met has extensively been shown [18,25]. We also detected binding of VEGF_165_ to ASs6 cells expressing CD44v6 and transfected with VEGFR-2 whereas AS cells expressing only VEGFR-2 (Figure 2B, left panel) showed a binding similar to uncoated control wells. In HUVECs used here as a positive control, binding of VEGF_165_ was detected. The co-receptor function of CD44v6 for VEGFR-2 was shown in these cells [15] and they express both CD44v6 and VEGFR-2.

Both in the case of HGF and VEGF_165_, the v6 peptide abrogated binding of the growth factors to ASs6 cells (Figures 2A and 2B right panels).

Taken together the FACS analysis and the cellular ELISA show that both HGF and VEGF_165_ bind only to CD44v6-expressing cells. In addition, in the case of Met and VEGFR-2, the FACS analysis revealed that the ligands are able to bind to cells even in the absence of their authentic receptor.

### A purified CD44v6 ectodomain inhibits HGF-induced migration and scattering

The preferential binding of HGF and VEGF to cells expressing CD44v6 suggests a direct interaction between CD44v6 and these growth factors. In order to test whether CD44v6 directly binds to HGF and VEGF and to measure binding affinities, we produced the rat CD44v6 ectodomain in HEK293T cells (Supplementary Figure S2). The hexahistidine-tagged CD44v6 ectodomain was purified by means of IMAC (immobilized metal ion affinity chromatography). The different fractions were screened in Western blot analysis with specific antibodies against CD44v6 and penta-histidine (His) (Supplementary Figure S2A). To confirm the CD44v6 sequence, the protein was digested with trypsin and analysed with UHPLC–MS/MS (Supplementary Figure S2B). Figure S2C lists all identified peptide sequences and their corresponding measured and theoretical masses and the calculated mass error. Since it was shown that three amino acids (EWQ) in the region of the variable exon 6 are essential for the function of CD44v6 as a co-receptor for Met [14] the peptide sequence containing these amino acids (highlighted in green) was further analysed with spectral interpretation of peptide fragments induced by CID. The peptide sequence WFENEWQGK could be confirmed via intact peptide mass spectra across two charge states (*z*=1 and *z*=2) and via their series of typical fragment series shown with *a* and *y* series (Supplementary Figure S2D).

**Figure 3:**
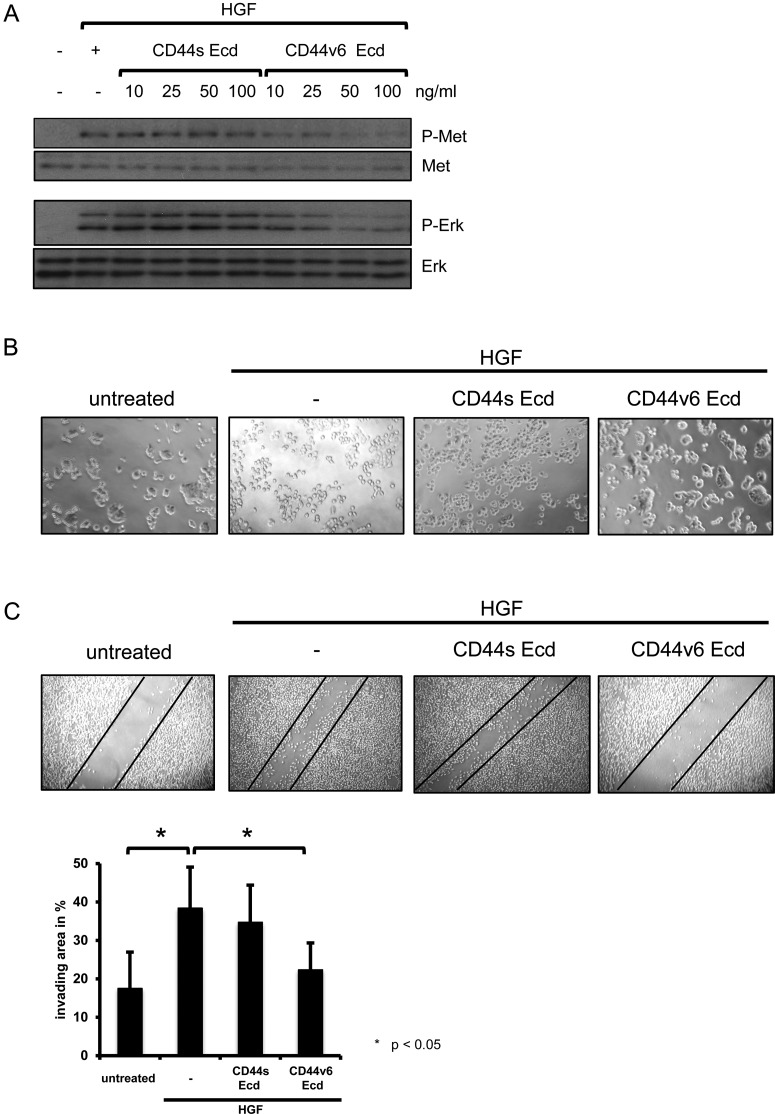
The CD44v6 ectodomain is functional after the IMAC purification (**A**) ASs6 cells were serum-starved, incubated with different concentrations of the CD44 ectodomains (labelled as Ecd) as indicated and induced with HGF. The cell lysates were separated by SDS-PAGE and subjected to Western blot analysis for detection of phosphorylated Met (P-Met) and phosphorylated Erk (P-Erk). For the loading control, the membrane was stripped and re-probed with specific antibodies against Met and Erk. The Western blots were performed at least three times and gave similar results. A representative blot is shown. (**B**) HT29 cells were serum-starved, incubated with the different CD44 ectodomains (25 μg/ml) as indicated and induced with HGF. 24 h after HGF-induction scattering of cells was observed with the Axiovert 40c Zeiss microscope (10× objective) and pictures were taken. (**C**) Representative pictures of monolayer wound assays performed with HeLa cells in the presence or absence of HGF, CD44s or CD44v6 as indicated. Top: Representative pictures taken 24 h after monolayer wounding. The black lines indicate the wound border immediately after monolayer wounding. Below: Quantification is represented as percentage of invaded area. Experimental data are reported as mean ± S.D. of three independent experiments (*P< 0.05; Student's *t*-test).

In order to test if the CD44v6 purified ectodomain was still active after the different purification steps, we tested its ability to block HGF-induced Met activation and HGF-induced scattering and migration in a monolayer-wounding assay (Figure 3). The transfected v6 containing CD44 ectodomain has been shown to compete with endogenous CD44v6 preventing the activation of Met [18]. Increasing amounts of CD44s ectodomains as well as CD44v6 ectodomains were added for 5 min to ASs6 cells prior to HGF induction and the activation of both Met and Erk was measured (Figure 3A). No effect on Met and Erk activation was observed with the CD44s ectodomain. In contrast, phosphorylation of Met and Erk decreased in a concentration dependent manner upon treatment with the CD44v6 ectodomain.

HGF induces dissociation of cells, epithelial to mesenchymal transition and enhanced migration, a process called scattering (reviewed in [26]). We tested the effect of the CD44s and CD44v6 ectodomains on HGF-induced scattering (Figure 3B). HT29 cells were incubated with the CD44s and CD44v6 ectodomains prior to HGF induction. Cells were observed 24 h after HGF treatment. Scattering was blocked by means of the CD44v6 ectodomain whereas no inhibitory effect was observed with the CD44s ectodomain. A migration assay was also performed. The migration of HeLa cells was measured in the presence or absence of the CD44v6 and CD44s ectodomains using monolayer-wounding assays (Figure 3C). HGF clearly induced migration of HeLa cells and closure of the wound in control plates. This effect was completely blocked by treatment of cells with the CD44v6 ectodomain whereas the CD44s ectodomain showed no effect.

Thus the monitoring of activation of Erk and Met as well as the scattering and the migration assays indicate that the CD44v6 ectodomain is functional and can be further used to study a potential direct binding to HGF and VEGF.

### Direct binding of the CD44v6 ectodomain to HGF and VEGF

**Figure 4:**
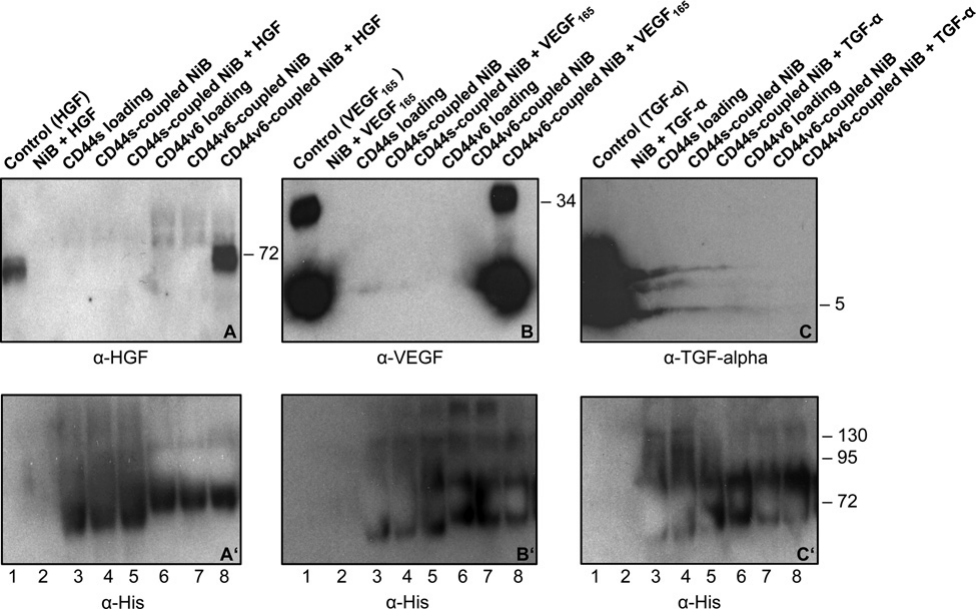
The CD44v6 ectodomain directly binds HGF and VEGF in pull-down assays (**A**–**C**') The nickel pull-down assays were performed with the histidine-tagged ectodomains of CD44 (CD44s and CD44v6). The CD44 ectodomains were coupled to Ni-NTA beads (labelled as NiB) and incubated with the growth factors HGF, VEGF_165_ and TGF-α as indicated. (**A**, **B**, **C**) Binding of the ligands to the histidine-tagged CD44 ectodomains was analysed in Western blots by means of specific antibodies against HGF, VEGF and TGF-α. As loading controls, the respective growth factors as well as the CD44 ectodomains were loaded directly on the gel. (**A**', **B**', **C**') To control the equal loading of the ectodomains the membranes were stripped and reprobed with a specific antibody against histidine for detection of the histidine-tagged CD44 proteins. The Western blots were performed at least three times and gave similar results. A representative blot is shown.

We then tested whether the purified CD44v6 ectodomain could directly bind to purified HGF or purified VEGF_165_ in a pull-down assay (Figure 4). To this aim, purified histidine-tagged CD44v6 ectodomain was pulled down by means of nickel-coated beads and subsequently incubated with HGF or VEGF_165_. TGF-α was used as a negative control since we have data indicating that the activation of EGFR by TGF-α is independent of CD44v6 (I. Morath, C. Jung and V. Orian-Rousseau, unpublished data). Blots 4A, B and C were incubated with antibodies against HGF, VEGF and TGF-α respectively. A strong signal was detected upon incubation of the CD44v6 ectodomain loaded nickel beads (Ni) with HGF or VEGF_165_ (last lanes in Figures 4A and 4B; incubation with the respective antibodies as indicated). In contrast, no such signal was seen in the case of TGF-α in the presence of nickel beads and of the CD44v6 ectodomain (Figure 4C). No signal was observed when HGF, VEGF_165_ or TGF-α was incubated with the nickel beads alone (2^nd^ lane in blots 4A, 4B and 4C, respectively) or with CD44s together with nickel beads (5^th^ lane in blots 4A, B and C, respectively). Also the CD44v6 ectodomain alone or in the presence of nickel beads gave no signal in the HGF, VEGF and TGF-α blots (6^th^ and 7^th^ lane in blots 4A, B and C, respectively) similarly to the CD44s ectodomain (3^rd^ and 4^th^ lane in blots 4A, 4B and 4C, respectively). The growth factors HGF, VEGF_165_ and TGF-α were loaded as controls directly on the gel in the first lane of blots 4A, B and C, respectively. In conclusion, a direct binding of HGF as well as VEGF_165_ to the CD44v6 ectodomain could be detected by means of pull-down assays. No binding of any growth factor was observed with the CD44s ectodomain. No binding was detected between TGF-α and the CD44v6 ectodomain. The same blots were also incubated with a penta-his antibody (Figure 4, lower blots A', B' and C') to visualize the histidine-tagged CD44 (CD44s and CD44v6) ectodomains.

### CD44v6 binds to HGF with low affinity and to VEGF with high affinity

**Figure 5:**
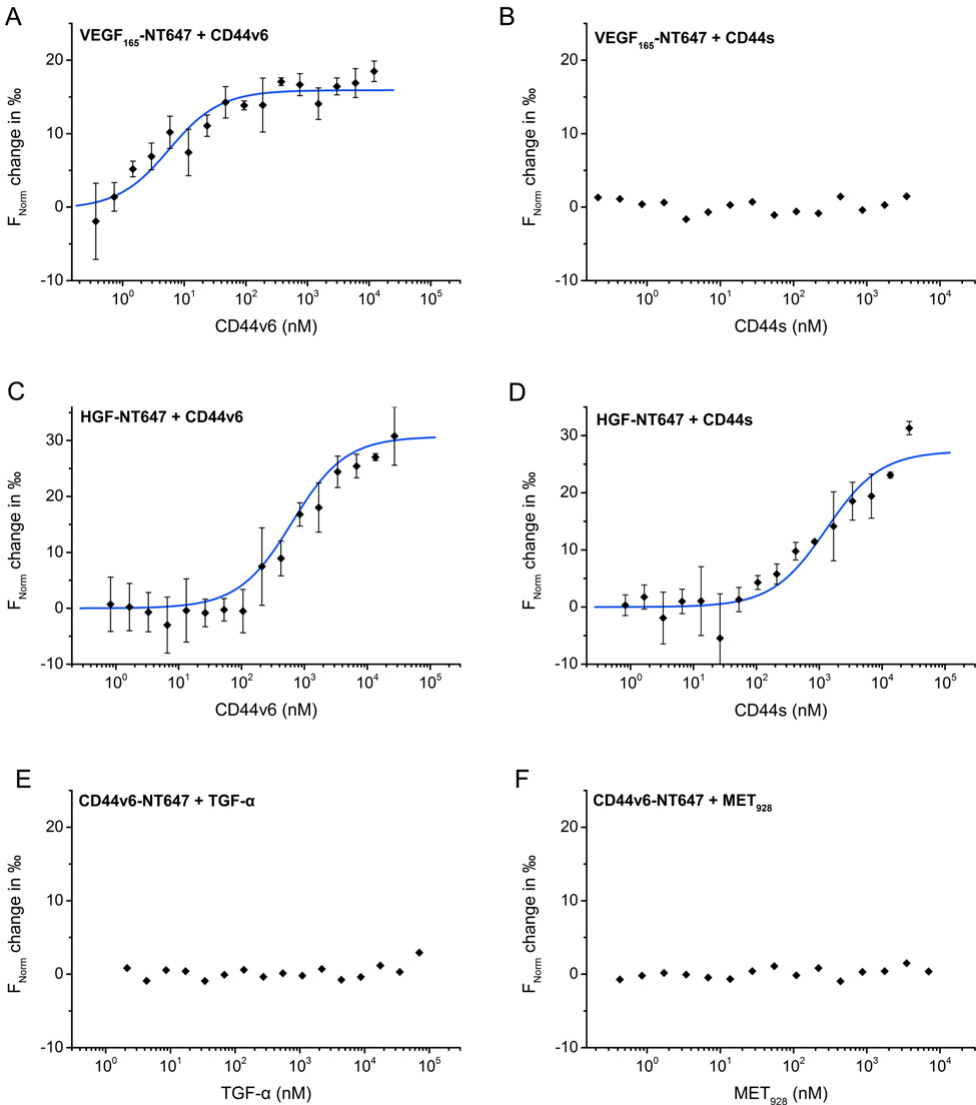
The CD44v6 ectodomain directly binds HGF and VEGF in MST (**A**–**F**) Using MST the *K*_d_ was determined whenever binding was observed (fitting curves in blue). (**A**) Fluorescently labelled VEGF_165_ was titrated against varying concentrations of the CD44v6 ectodomain (*K*_d_=51±9 nM) or (**B**) the CD44s ectodomain. (**C**) Titration of serial dilutions of the CD44v6 ectodomain (*K*_d_=610±90 nM) or (**D**) the CD44s ectodomain (*K*_d_=130±300 nM) to fluorescently labelled HGF. (**E**) Fluorescently labelled CD44v6 ectodomain was titrated against serial dilutions of TGF-α or (**F**) against the Met ectodomain (Met_928_). Binding affinities were calculated from at least three different experiments.

In order to define the dissociation constant (*K*_d_) of the binding between HGF and the CD44v6 ectodomain as well as VEGF_165_ and the CD44v6 ectodomain, we used MST (Figures 5A–5D). Compared with other methods like ELISA or SPR (surface plasmon resonance), MST offers the advantage that the binding is measured in solution. No immobilization of one binding partner is necessary. The mobility of a fluorescently labelled molecule is measured in a microscopic temperature gradient. Fluorescently labelled HGF or VEGF_165_ was incubated with serial dilutions of the CD44v6 ectodomain. A clear binding was observed between VEGF_165_ and the CD44v6 ectodomain (Figure 5A) and also between HGF and the CD44v6 ectodomain (Figure 5C). The *K*_d_ for the binding of HGF to CD44v6 was 610±90 nM whereas a substantially higher affinity of 51±9 nM was observed between VEGF_165_ and CD44v6. To our surprise, binding was observed between unlabelled CD44s and the fluorescently labelled HGF (Figure 5D) but not between CD44s and the fluorescently labelled VEGF_165_ (Figure 5B). However, no binding at all was observed between TGF-α and fluorescently labelled CD44v6 (Figure 5E). No binding of Met_928_ to labelled CD44v6 was observed (Figure 5F).

**Figure 6:**
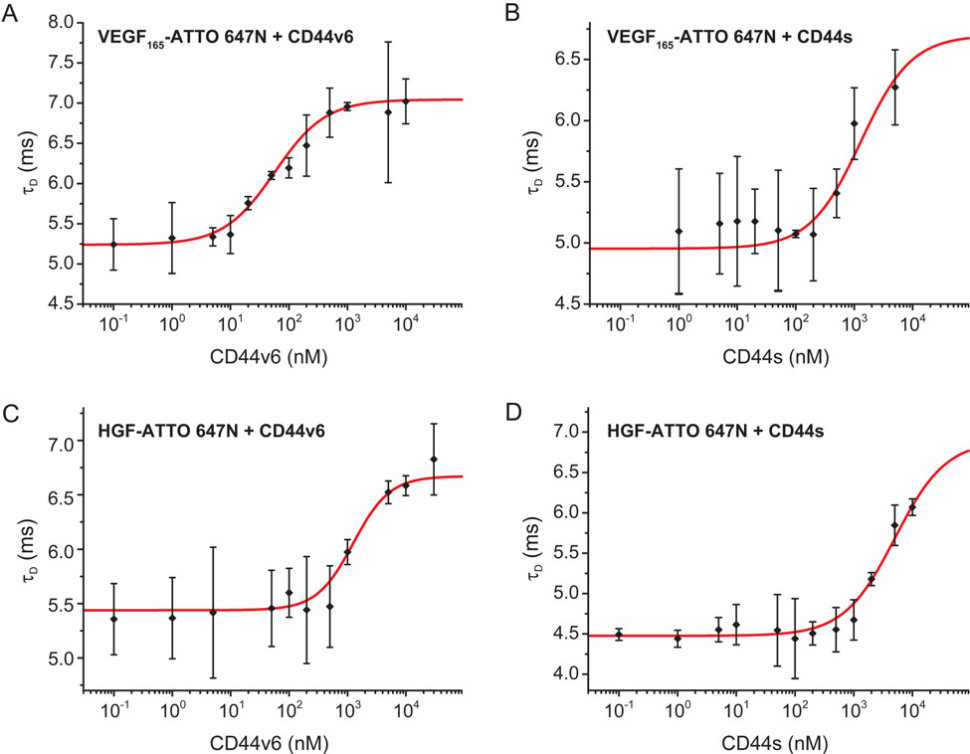
FCS binding curves of CD44 isoforms with HGF and VEGF (A–D) Dissociation constants were determined via FCS by titrating the respective CD44 isoform against the fluorophore-labelled growth factor. The study on VEGF_165_-ATTO 647N binding to (**A**) CD44v6 and (**B**) CD44s revealed dissociation constants of 65±8 nM and 1300±600 nM, respectively. The same experiment was conducted with HGF-ATTO 647N yielding a (**C**) *K*_d_=1830±60 nM for CD44v6 and (**D**) *K*_d_=5000±3000 nM for CD44s.

Two other methods, namely FCS (Figure 6) and FA (Supplementary Figure S3) were used to test the binding of CD44v6 to HGF and VEGF. In FCS, fluctuations in the fluorescence signal due to the diffusion of fluorophore-labelled proteins in and out of the focal volume are monitored. By analysing changes in the diffusion time, the binding affinity can be determined. Using this well-established method, we measured the binding of HGF-ATTO 647N and VEGF_165_-ATTO 647N to CD44v6 and obtained dissociation constants of 1830±64 (S.D.) nM and 65±8 (S.D.) nM, respectively. These results and binding affinities are consistent with the MST measurements. In the case of HGF, CD44s was shown to bind in the same range (5000±3000 nM) as CD44v6. In contrast, the binding affinity of VEGF_165_ to CD44s (1300±600 nM) was significantly reduced compared with CD44v6.

In fluorescence anisotropy, the polarized light of the fluorophore emission is used to determine the binding constants. By adding increasing concentrations of unlabelled CD44v6 to NT647-labelled HGF (Supplementary Figure S3A), we observed an increase in anisotropy, indicative of the slower rotation of the HGF molecules as a result of their increase in size due to their binding with CD44v6. FA confirmed the direct interaction between HGF and CD44v6 with about the same low affinity (3700±600 nM) as measured in FCS. An increased anisotropy was also observed for CD44s (Supplementary Figure S3B) (10,000±8000 nM). The affinity is here lower than the one measured between CD44v6 and HGF.

When looking at the measured binding affinities across all methods (Table 1), VEGF_165_ shows a strong binding to CD44v6 while HGF binds with a lower affinity. The calculated binding affinity for the CD44s ectodomain to HGF is in the same order of magnitude as for CD44v6 but is consistently 2–3 folds lower in all three methods.

**Table 1:** Overview of binding affinities as determined with MST, FCS and FA The dissociation constants (*K*_d_) shown are the average values of *N* experiments carried out for each pair of binding partners.

Method	Labelled protein	Binding partner	*K*_d_	*N*
MST	HGF-NT647	CD44v6	610±90 nM	5
FCS	HGF-ATTO 647N	CD44v6	1830±60 nM	6
FA	HGF-NT647	CD44v6	3700±600 nM	3
MST	HGF-NT647	CD44s	1300±300 nM	3
FCS	HGF-ATTO 647N	CD44s	5000±3000 nM	1
FA	HGF-NT647	CD44s	10000±8000 nM	2
MST	VEGF_165_-NT647	CD44v6	51±9 nM	3
FCS	VEGF_165_-NT 647	CD44v6	200±40 nM	2
FCS	VEGF_165_-ATTO 647N	CD44v6	65±8 nM	2
MST	VEGF_165_-NT647	CD44s	No fit	3
FCS	VEGF_165_-ATTO 647N	CD44s	1300±600 nM	1
MST	CD44v6-NT647	TGF-α	No fit	3
MST	CD44v6-NT647	Met_928_	No fit	2

To address the mode of action of the CD44v6 peptide used in FACS analysis and cellular ELISA, we tested whether the CD44v6 peptide directly binds to the CD44v6 ectodomain (Supplementary Figure S4A) or HGF (Supplementary Figure S4B). In MST, the CD44v6 peptide could bind the CD44v6 ectodomain with a high affinity of 2.10±0.2 nM. However no binding could be detected between the CD44v6 peptide and HGF (Supplementary Figure S4B).

## DISCUSSION

CD44v6 has been shown to act as a co-receptor for Met and VEGFR-2 in various types of primary cells including keratinocytes [18] and HUVECs [15] and in several cancer cell lines (reviewed in [12]). The CD44v6 ectodomain is required for Met and VEGFR-2 activation whereas the cytoplasmic domain connected to the cytoskeleton through ERM proteins, allows signalling. In the present paper, FACS analysis revealed binding of HGF and VEGF_165_ only to cells expressing CD44v6. HGF binding to CD44v6-expressing cells could be competed against by addition of soluble CD44v6. The binding of VEGF_165_ and HGF to cells could be blocked by v6 peptides. These data suggest that the binding of these growth factors to CD44v6 is mediated by interaction with the protein part of CD44v6 and not with protein-linked sugars. A complementary method, namely cellular ELISA, confirmed these findings. Pull-down assays using purified CD44v6 ectodomain and HGF qualitatively showed a direct interaction of these two proteins in a binary system. The same is true for VEGF_165_ and CD44v6. These bindings were confirmed and quantified by MST, FCS and FA measurements. Intriguingly, although no binding could be observed between CD44s and HGF in pull-down assays, a binding could be measured in solution in MST, FCS and FA. This binding between HGF and CD44s is consistently in all methods lower than the one measured between CD44v6 and HGF. In the case of VEGF_165_, no binding to CD44s was observed in MST whereas CD44s bound to VEGF_165_ with a twenty times lower affinity as compared with CD44v6 in FCS.

Although the overall behaviour of CD44v6 as a co-receptor for Met and VEGFR-2 is very similar, a clear difference could be seen between the binding of CD44v6 to HGF and to VEGF. The binding of VEGF_165_ to purified CD44v6 ectodomain is a high affinity binding. This binding is specific to CD44v6 since CD44s binds VEGF_165_ with a 20 times lower affinity. It is therefore possible that the binding of VEGF to CD44v6 is a crucial step in the activation of VEGFR-2 by VEGF. Moreover, the direct binding of VEGF_165_ to a non-HS form of CD44, namely CD44v6 would indicate that heparin/HS, if required, is not provided by CD44. The influence of heparin/HS on the binding of VEGF_165_ to VEGFR-2 was demonstrated in endothelial cells where heparin/HS was shown to enhance binding of ^125^I-VEGF_165_ to VEGFR-2 [27]. The neuropilins that have also been described as co-receptors for VEGFR-2 interact with heparin/HS [27]. Sprouting angiogenesis is sensitive to HS degradation [28]. Moreover a model where HSPGs acting in trans might regulate VEGFR-2 signalling was proposed. In the same line, VEGF-dependent guidance of vascular sprouts was shown to involve HSPGs [29]. Taken together, these data suggest a complex between CD44v6, VEGF, VEGFR-2 and neuropilin that would promote angiogenesis.

In contrast with VEGF_165_, the binding affinity measured *in vitro* for HGF and the CD44v6 ectodomain is in the micromolar range. This is in contrast with the high affinity binding of HGF to a soluble Met receptor fusion protein measured in the picomolar range [30] and nanomolar range [31]. More precisely it was shown that the α-chain of HGF binds to Met with high affinity [30] and the β-chain with lower affinity [32]. That CD44v6 binds HGF with low affinity is particularly surprising since the binding of HGF to Met-expressing cells is strictly dependent on the presence of CD44v6. Low-affinity binding of HSPGs to growth factor has been shown to cluster growth factors and to increase their local concentrations in the vicinity of their authentic receptors. The accumulation of FGF-2 close to FGFR-2 promoted sustained activation of the FGF pathway in endothelial cells [33]. Such a low-affinity binding site has been extensively described in the case of HSPGs such as syndecans that are essential for the clustering of FGF-2 via heparin/HS binding and its presentation to FGFR-1 [3]. Elimination of HS from the cell surface reduced binding of FGF-2 to FGFR-1 [34]. However, the CD44v6 isoform shown to act as a co-receptor for Met does not contain exon v3, the only known HS attachment site in CD44 [11].

Discrepancy between *in vivo* data measured on cells and *in vitro* data obtained with purified proteins are also observed in the case of Met. Indeed, as mentioned above purified Met binds HGF with high affinity whereas our cellular assays suggest that HGF does not bind Met-expressing cells in the absence of CD44v6. These discrepancies might be due to the fact that the *in vitro* binding experiments investigating the binding of HGF to Met were performed exclusively with a purified Met ectodomain [30,35] and not with a full-length Met molecule. Membrane-bound Met might exhibit lower affinity for HGF than the isolated Met ectodomain, e.g. due to an inhibitory effect of the transmembrane or the cytoplasmic domain. In the literature, there are several examples of receptors, in which the cytoplasmic region influences ligand affinity of the extracellular domain by conformational coupling via the transmembrane domain. In integrins, association of the cytoplasmic domains stabilizes the extracellular domain in a low-affinity state [36]. Even addition of only the transmembrane domains to a recombinant integrin ectodomain lowered the ligand affinity [37]. In the case of epidermal growth factor receptor (EGFR), negative cooperativity for binding of ligands to the extracellular domain is only observed for the intact transmembrane protein, but not upon deletion of the cytoplasmic part or for the soluble ectodomain [38].

Binding of HGF to Met might depend on the association of HGF or Met to a co-receptor such as CD44v6. However, a direct effect of CD44v6 on Met in order to alleviate such an auto-inhibition seems unlikely, as the interaction of the two proteins in the cell depends on the presence of HGF. In addition, direct contact between the purified Met_928_ ectodomain and CD44v6, could not be shown by MST. Thus HGF might be required to bridge CD44v6 and Met. Indeed, the Met/CD44v6 complexes detected upon immunoprecipitation were obtained only in the presence of HGF [13,18,19].

CD44s cannot act as a co-receptor for Met, cannot support the binding of HGF to cells and cannot be pulled down with HGF. However binding of CD44s to HGF was observed in MST, FCS and FA. We therefore have to consider whether CD44v6 has additional functions for Met that CD44s cannot fulfil. What other role could CD44v6 play? CD44v6 might participate in the dimerization of Met. The Met dimer structure is not known and till now it was not possible to show how HGF can induce dimerization of Met itself *in vitro* although several attempts were made using a purified Met ectodomain and recombinant HGF (reviewed in [39]). Only a short part of the Met ectodomain was shown to form a 2:2 complex in the presence of an HGF dimer while Met_928_ did not [40]. That HGF could not induce Met dimerization might be due to the fact that the Met truncated version used for these assays lacked essential sequences for dimerization. For example it is known that the transmembrane domain can stabilize the dimers of several RTKs (reviewed in [41]). Dimerization of Met might require other molecules than HGF. CD44v6 might be such a candidate since it has been shown to be required for Met phosphorylation, a direct consequence of the dimerization of the intracellular tyrosine kinase domains of Met. It is possible that CD44v6 and not CD44s allows Met to enter a stable homodimer configuration. While both CD44v6 and CD44s had binding affinities to HGF in the same order of magnitude, MST, FCS and FA all presented a 2–3 folds difference that might be relevant for interactions with HGF *in vivo*. As we have shown in this work, HGF is able to directly bind CD44v6 and Met and thus HGF might bridge the two transmembrane proteins to allow a protein complex arrangement necessary for Met dimerization. In addition, the binding of CD44v6 to HGF might stabilize the very weak dimerization of HGF itself, which is speculated to be the prerequisite for Met dimerization [40].

Of note, the CD44v6 peptide prevented the binding of growth factors to cells in FACS analysis and cellular ELISA. Since it binds to the CD44v6 ectodomain but not to HGF, a possible mode of action of this peptide is a competition with HGF for binding to CD44v6. Indeed, we showed that the affinity of the CD44v6 peptide for the CD44v6 ectodomain is several magnitudes higher than the affinity of HGF for the CD44v6 ectodomain. The high affinity of the CD44v6 peptide for the CD44v6 ectodomain might explain its high efficiency in inhibition of tumour angiogenesis [15].

In conclusion, a non-HS isoform of the CD44 family, CD44v6 directly binds to HGF and VEGF. The difference between the binding affinities measured for HGF/CD44v6 and VEGF_165_/CD44v6 and the fact that CD44s binds HGF but not VEGF_165_ might however reflect a different mode of action between Met and VEGFR-2 co-receptor function of CD44v6.

## Abbreviations

AS: rat pancreatic carcinoma BSp73AS cells
ASs6: BSp73ASs6/CD44v6-transfected AS cells
CAM: cell adhesion molecule
CID: collision-induced dissociation
Ecd: ectodomain
FA: fluorescence anisotropy
FCS: fluorescence correlation spectroscopy
FGF-2: fibroblast growth factor 2
FGFR: fibroblast growth factor receptor
HGF: hepatocyte growth factor
HRP: horseradish peroxidase
HS: heparan sulfate
HSPG: heparan sulfate proteoglycan
HUVEC: human umbilical vein endothelial cells
HT29: human colon adenocarcinoma cells
*K*_d_: dissociation constant
MST: MicroScale Thermophoresis
Ni-NTA: nickel-nitrilotriacetic acid
NRP-1/2: neuropilin 1 and 2
PE: phycoerythrin
RTK: receptor tyrosine kinase
PLL-PEG: poly-L-lysine polyethylene glycol
VEGFR-2: vascular endothelial growth factor receptor 2
TGF-α: transforming growth factor alpha
VEGF_165_: vascular endothelial growth factor A_165_
